# Supplementary material for: Splice-Junction-Based Mapping of Alternative Isoforms in the Human Proteome
Source: Cell Rep. Author manuscript; Available in PMC 2020 Jan 15. (PMC6961840; doi:10.1016/j.celrep.2019.11.026)
Supplement: 3 [file NIHMS1546469-supplement-3.zip › DF2/PXD0010154/Liver-110-P11171-KEDEPPEQAEPEPTEAWKK.pdf]

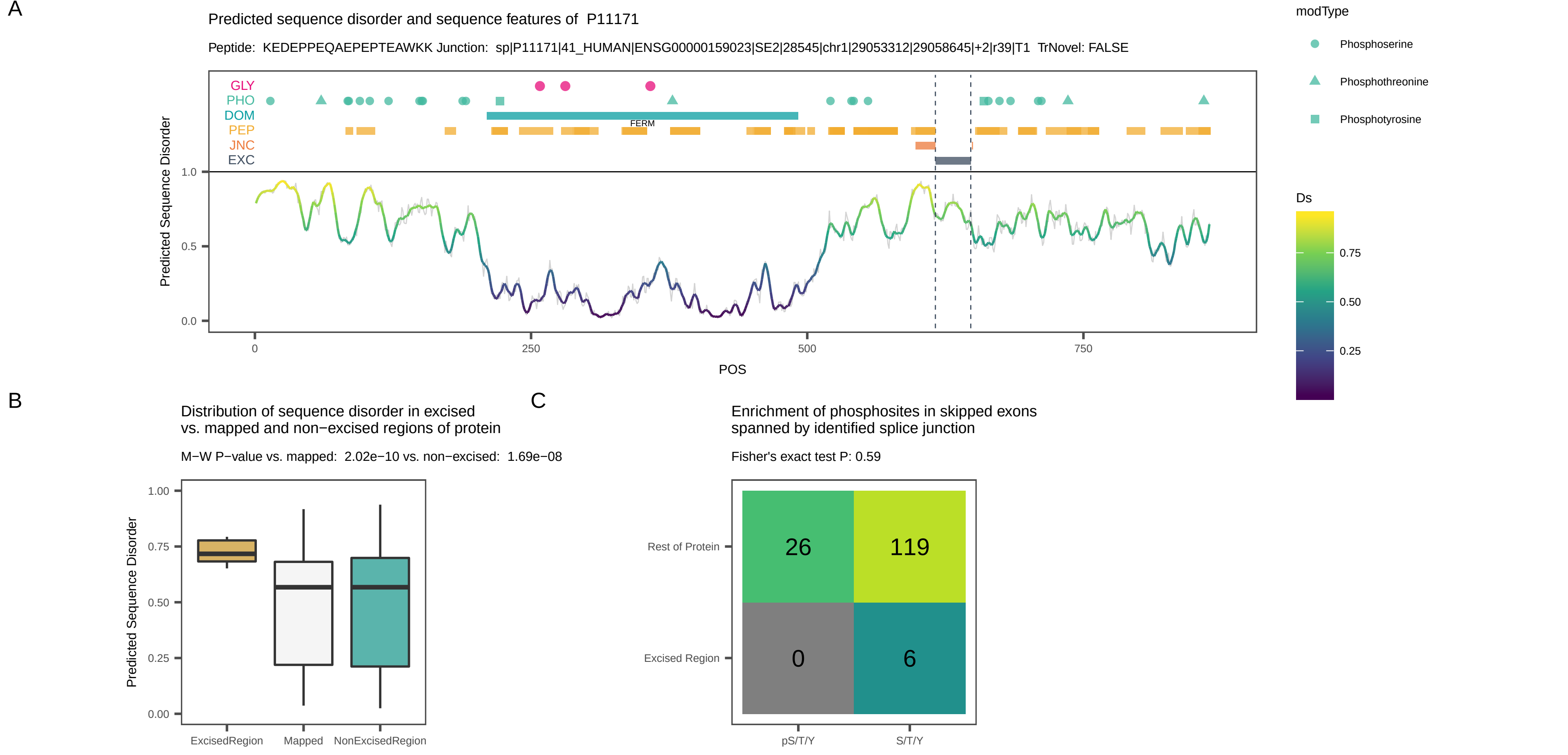

A
Predicted sequence disorder and sequence features of P11171
Peptide: KEDEPPEQAEPEPTEAWKK Junction: sp|P11171|41_HUMAN|ENSG00000159023|SE2|28545|chr1|29053312|29058645|+2|r39|T1 TrNovel: FALSE
GLY
PHO
DOM
PEP
JNC
EXC
FERM
Predicted Sequence Disorder
1.0
0.5
0.0
0
250
500
750
POS
modType
Phosphoserine
Phosphothreonine
Phosphotyrosine
Ds
0.75
0.50
0.25
B
Distribution of sequence disorder in excised
vs. mapped and non−excised regions of protein
M−W P−value vs. mapped: 2.02e−10 vs. non−excised: 1.69e−08
Predicted Sequence Disorder
1.00
0.75
0.50
0.25
0.00
ExcisedRegion
Mapped
NonExcisedRegion
C
Enrichment of phosphosites in skipped exons
spanned by identified splice junction
Fisher's exact test P: 0.59
Rest of Protein
Excised Region
26
119
0
6
pS/T/Y
S/T/Y
